# Post-COVID Syndrome and Cardiorespiratory Fitness—26-Month Experience of Single Center

**DOI:** 10.3390/life13030684

**Published:** 2023-03-02

**Authors:** Milan Sova, Eliska Sovova, Jaromir Ozana, Katarina Moravcova, Marketa Sovova, Libor Jelinek, Jan Mizera, Samuel Genzor

**Affiliations:** 1 Department of Respiratory Medicine, Faculty of Medicine and Dentistry, University Hospital Olomouc, Palacky University Olomouc, 779 00 Olomouc, Czech Republic; 2 Department of Respiratory Medicine, Faculty of Medicine and Dentistry, University Hospital Brno, Masaryk University Brno, 601 77 Brno, Czech Republic; 3 Department of Sport Medicine and Cardiovascular Rehabilitation, University Hospital Olomouc, Palacky University Olomouc, 779 00 Olomouc, Czech Republic; 4 Center for Digital Health, University Hospital Olomouc, Palacky University Olomouc, 779 00 Olomouc, Czech Republic

**Keywords:** post-COVID syndrome, cardiopulmonary exercise testing

## Abstract

Introduction: Persistent post-COVID syndrome, also referred to as long COVID, is a pathologic entity that involves persistent physical, medical, and cognitive sequelae following COVID-19. Decreased fitness has repeatedly been reported in numerous studies dealing with post-COVID syndrome, however, it is still not fully clear which groups of patients may be more susceptible for persisting symptoms. Aim: The aim of our study was to evaluate the number of post-COVID patients with cardiac symptoms, where these patients were evaluated by CPET and the results compared with a control group of patients. Methods: Follow-up of patients in post-COVID outpatient clinic from 1 March 2020 to 31 May 2022. Inclusion criteria were positive PCR test for SARS-CoV-2 and age 18–100. The initial examination was performed 4–12 weeks after the disease onset. All patients with possible cardiac symptoms had completed cardiopulmonary exercise testing. The control group was randomly selected from a database of clients in 2019, with the preventive reason for evaluation. Results: From 1 March 2020 to 31 May 2022, 2732 patients (45.7% males) were evaluated with a mean age of 54.6 ± 14.7. CPET was indicated only in 97 patients (3.5%). Seventy-four patients (26 male) achieved the exercise maximum and a comparison were made with a control group (same age (*p* = 0.801), BMI (*p* = 0.721), and sex ratio). No significant dependence between the parameter VO_2_ max mL/kg/min and post-COVID disability was demonstrated (*p* = 0.412). Spearman’s correlation analysis did not show a significant relationship between the parameter VO_2_ max mL/kg/min and the severity of COVID-19 (*p* = 0.285). Conclusions: Cardiac symptoms occurred in only a small percentage of patients in our study. There is a need for further studies that would objectively evaluate the effect of COVID-19 disease on the patient’s health.

## 1. Introduction

The COVID-19 (coronavirus disease 2019) pandemic affected the whole world and led to the death of millions of people and to the introduction of hygiene measures including lockdowns with great economic, psychological, and other consequences. Gradually, with the development of the pandemic, there were also cases of cured patients whose symptoms persisted even after the acute illness had subsided. The nomenclature of this syndrome is still inconsistent, and the name long COVID-19 or post-COVID-19 syndrome is used. This syndrome occurs in people (in children, adolescents, and adults) who have experienced this disease with both a severe and mild course. It is defined as a set of symptoms persisting for more than 12 weeks or longer after the diagnosis of COVID-19 and which cannot be explained in any other way [1]. Post-COVID complications are defined as: (a) symptoms that persist, worsen, recur, or re-emerge in relation to the acute infection; (b) deterioration in the quality of life or functional status compared to the period before experiencing COVID-19; and (c) the presence of otherwise inexplicable persistent or progressing pathological organ findings during imaging, laboratory, or functional examinations [1]. Typical problems are long-lasting fatigue, shortness of breath, deterioration of mental functions, pain in the chest, joints, palpitations, muscle pain, taste and smell disorders, headaches, hematological, and gastrointestinal problems [2]. Pathophysiological mechanisms have not yet been precisely described, however, the role of direct tissue involvement (e.g., lung, heart, brain) and pathological inflammation (virus persistence, immune dysregulation, autoimmunity) have been reported. During COVID-19 infection, there is an excessive development of systemic inflammatory response syndrome (SIRS) with high levels of pro-inflammatory cytokines. The organism reacts by developing compensatory anti-inflammatory response syndrome (CARS) in an attempt to achieve balance. However, if this anti-inflammatory response is inadequately high, prolonged immunosuppression, known as PICS (persistent inflammation, immunosuppression, catabolic syndrome), occurs. This is one of the most considered causes of post-COVID complications. Patients are at increased risk of bacterial and fungal infections and are also prone to developing pulmonary fibrosis. Female sex, early dyspnea, previous psychiatric illnesses, and the presence of specific biomarkers (D dimers, CRP, lymphocyte count) are described as risk factors [3]. The time limit is usually given as symptoms lasting more than 4 or 12 weeks after the infection [4], with some studies even reporting up to 24 weeks [5]. The frequency of occurrence of this syndrome has not yet been determined; according to studies, it occurs in 4.7–80% of patients after the disease of COVID-19 [5]. Some meta-analyses report, for example, the occurrence of fatigue after the disease in up to 32% of patients, disorders of mental functions in 22% of patients [6], others, for example, 63.2, 71.9, and 45.9% occurrence of more than one post-COVID symptom at 30, 60, or more than ≥90 days after hospitalization. The most frequently described symptoms are fatigue and shortness of breath as well as persistent cough (20–25%), smell disorders (10–20%), taste disorders (15–20%), or joint pain (15–20%) [7]. In some patients after COVID-19 disease, changes on the CT of the lungs persist, which mainly include opacities of the milk glass and the development of pulmonary fibrosis. Predictors of this disability are mainly mechanical ventilation, stay in an intensive care unit, high inflammatory markers, long hospitalization, and the presence of acute respiratory distress syndrome [8,9].

A study by Pavli et al. [10] estimated the prevalence of post-COVID symptoms to be 10–35% in non-selected individuals. For hospitalized patients, it may reach up to 85% of individuals. Fatigue was identified as the most common symptom reported in 17.5–72% of post-COVID cases, followed by dyspnea with an incidence of 10 to 40%. Mental disorders, thoracalgia, olfactory, and gustatory disorders may be present in up to 26, 22, and 11% of patients, respectively. Many patients with persisting post-COVID symptoms have at least one pre-existing condition or comorbidity, which can definitely influence the presence of the accompanied symptoms.

In a recent study by Genzor et al. [11], we evaluated the post-COVID symptoms in post-acute COVID-19 individuals (*N* = 785). Dyspnea was present more frequently in more severe forms and persisted in 40% of patients after mild COVID-19, which was significantly less than 63.7% after aa severe COVID-19 course. Pulmonary function tests in the study were also significantly reduced in more severe forms, especially diffusing capacity (mean of 86.3 in the mild COVID group, compared with a mean 68% in the severe COVID group).

One of the largest available studies was questionnaire-based with 76,422 participants, of these, 4231 (i.e., 5.5% participants) had COVID-19 and were matched to 8462 healthy controls. A total of 12.7% of them presented symptoms that could be attributed to COVID-19 [12].

All of these pathological changes are possibly leading to increased mortality and a significantly reduced quality of life in affected individuals [13]. The scientific community is still looking for the exact etiopathogenesis and clinical development of these “post-COVID” complex symptoms. However, many of them are actually very close to symptoms typically observed in chronic fatigue syndrome [14].

Cardiac symptoms of long COVID may include chest pain, shortness of breath, fatigue, and signs of autonomic dysfunction such as postural orthostatic tachycardia. There is a large discrepancy between symptom severity and the objective assessment of cardiac function [15].

Assessing cardiorespiratory fitness is a clinical vital sign [16,17] and cardiopulmonary exercise testing (CPET) provides the most standardized quantification of CRF. Put simply, without CPET, the pathophysiologic impacts of COVID-19 that only manifest during physical exertion, or manifest more profoundly during physical exertion, would be missed entirely, preventing a holistic understanding of the clinical presentation [18]. Despite the extremely large numbers of studies dealing with COVID-19 and post-COVID syndrome, studies analyzing cardiorespiratory functions in post-COVID individuals are not as numerous. The vast majority of studies have confirmed that more severe COVID-19 infection leads to a more pronounced reduction in the exercise capacity [19,20,21,22,23,24,25,26,27,28,29,30]. Moreover, in all of the published studies, only a small number of patients were involved, and usually the involved individuals had survived more severe infection forms, or even individuals who were not discharged from the hospital. The growing scientific evidence shows that post-COVID syndrome may cause exercise intolerance, but the relationship between SARS-CoV-2 and exercise capacity remains unclear. Understanding the mechanism behind the limitation in exercise capacity is a fundamental step in improving patient outcomes. A hallmark of exercise intolerance is dyspnea and fatigue upon exertion. Exercise is dependent on the balance between oxygen supply, oxygen consumption, and the clearance of toxic metabolites. These processes rely on the cardiovascular and pulmonary systems to achieve optimal exercise performance. Cardiopulmonary exercise testing offers the opportunity to study the cellular, cardiovascular, and ventilatory systems’ responses simultaneously, providing an objective evaluation of exercise capacity and cardiorespiratory fitness. The aim of our study was to evaluate the number of post-COVID patients with cardiac symptoms, these patients were evaluated by CPET and the results compared with a control group of patients.

## 2. Methods

### 2.1. Participants

Follow-up of patients was carried out in a post-COVID outpatient clinic from 1 March 2020 to 31 May 2022. The inclusion criteria were positive polymerase chain reaction test for SARS-CoV-2 and age between 18 and 100. The initial examination was performed 4–12 weeks after the disease onset. All subjects underwent physical examination, history, chest X ray examination, and pulmonary function tests (including body-plethysmography and carbon monoxide diffusion measurement). If the patient had possible cardiac symptoms, it was evaluated in the Department of Sports Medicine (Cardiology Department was a COVID unit in this time) and CPET was carried out. The performance time of CPET was 12–15 weeks from the acute COVID-19 infection. Exclusion criteria for CPET study evaluation was the presence of active respiratory infection, non-compensated heart failure, recent or acute pulmonary embolism, acute or 4 weeks ago acute coronary syndrome, decompensated arterial hypertension, severe heart valvular disease, neuromuscular disease, and psychiatric disease making CPET impossible. The last exclusion criterion was significant pulmonary involvement indicated for the systemic corticosteroid treatment, as those individuals usually improve rapidly in time. The control group was randomly selected from a database of clients in 2019, with a preventive reason for evaluation. Among them, none had any serious comorbidity, and the results of CPET did not show any susceptibility of cardiac or other disorders.

### 2.2. Cardiopulmonary Exercise Testing

Cardiopulmonary exercise testing (CPET) was performed at the Department of Sports Medicine on an electromagnetically braked bicycle ergometer (Ergoline-Ergoselect 200; manufacturer: Ergoline GmbH, Bitz, Germany) using indirect calorimetry (Jaeger OXYCON pro) for continuous measurement of ventilation, oxygen consumption, expired carbon dioxide, with continuous 12 lead ECG monitoring. Blood pressure was measured manually in two minute intervals with oxyhemoglobin saturation. A modified Bruce protocol was used to the exercise maximum (RER over 1.05 and plateau in oxygen consumption). The resting and peak values of the monitored parameters have been marked: heart rate (HR), systolic and diastolic arterial blood pressure (SBP, DBP), respiratory equivalent (RER), maximal oxygen consumption (VO_2_ max mL, VO_2_ max mL/kg/min), ventilation/carbon dioxide production slope (VE/VCO_2_ slope), performance in watts (W), and in W/kg and metabolic equivalent (MET). Cardiovascular fitness (VO_2_ max, VO_2_ max/mL/kg/min) was correlated with the post-COVID chest x-ray findings and with clinical course (home, mild, home-pneumonia, hospitalization without oxygen support, hospitalization with oxygen support, hospitalization with high-flow nasal oxygen therapy, or non-invasive ventilation, hospitalization with invasive ventilation support, hospitalization with ECMO). All CPET examinations were performed with continuous presence of the physician for safety reasons.

### 2.3. Statistical Methods

For data analysis, we used statistical software IBM SPSS Statistics version 23 (IBM Corp., Armonk, NY, USA). To compare the quantitative parameters, we was used the Mann–Whitney U-test, and qualitative parameters were compared using Fisher’s exact test. To evaluate the correlation between VO_2_ max and post-COVID involvement, BMI and age, we used the Spearman’s correlation analysis. All tests were performed at the level of significance of 0.05. The normality of the distribution was evaluated using the Shapiro–Wilk test.

The study was in concordance with the Declaration of Helsinki and was approved by the local Ethics Committee; decision number EK 98/21. All individuals involved in the study participated voluntarily and signed their informed consent before the examination started.

## 3. Results

From 1 March 2020 to 31 May 2022, 2732 patients (1248 (45.7%) male) were evaluated with an average age of 54.6 ± 14.7, in which the males were 56.0 ± 14.5, and females were 53.4 ± 14.7. The basic study group characteristics are listed in Table 1. The results showed a normal distribution according to the Shapiro–Wilk test.

Prevalence of obesity was similar in both the controls and study group individuals (see Table 2). The proportion of obese individuals was without statistical difference. This is in concordance with a comparison of the BMI between the groups, which also showed no statistical differences (see Table 3).

CPET was performed in ninety-seven patients (3.5%), among them seventy-four patients (26 male) achieved the exercise maximum. The comparison was made with a control group (same age (*p* = 0.801), BMI (*p* = 0.721) and sex ratio). Among the post-COVID individuals, two patients (2.70%) had pneumonia without hospital admission, one patient (1.35%) hospitalization without the need of oxygen support, two patients had (2.70%) hospitalization with the need for oxygen support, two patients had hospitalization with the need for high-flow nasal oxygen support or non-invasive ventilation (2.70%), and the rest had survived the COVID-19 infection without the need of hospital admission and without pneumonia. Eleven patients (14.8%) had persisting chest-x-ray changes consistent with an organizing pneumonia pattern.

The most common persisting symptoms were dyspnea (79.7%), chest pain (25.6%), and palpitations (29.7%).

Nine patients (12.16%) had a history of arterial hypertension, seven (9.45%) had asthma, four (5.40%) had hypothyroidism (well compensated on the treatment), and two (2.70%) had cardiac arrhythmias (atrial fibrillation). Other serious comorbid diseases were not present.

CPET testing ended with hypertension reaction in eleven patients (14.86%), seven patients (9.45%) had ventricular or atrial premature beats, and one patient (1.35%) had chronotropic incompetence (due to usage of betablockers). None of the individuals had exercise induced bronchospasm during the examination, none of them ended the examination because of chest-pain, and none of them had susceptibility for ischemic heart disease.

A summary of the comparison of monitored parameters is in Table 3.

There was no significant difference between the patients and the control group in the resting blood pressure, resting heart rate, maximal systolic blood pressure, and maximal heart rate. Significantly lower values of the max diastolic blood pressure parameter were found in the patients (*p* = 0.003). No significant difference in the VO_2_ max and VO_2_ max mL/kg/min was found between the patients and the control group. Significantly lower values of respiratory exchange ratio (*p* = 0.0003), power in Watts (*p* = 0.048), and W/kg (*p* = 0.039) parameters were found in patients after COVID-19 compared to the control group.

Men differed significantly only in the max diastolic BP parameter; lower values were found in the group of patients (*p* = 0.049). In women in the patient group, compared to women in the control group, significantly lower values of diastolic BP max (*p* = 0.023), RER (*p* = 0.001), W (*p* = 0.008,) and W/kg (*p* = 0.048) were demonstrated.

### 3.1. Correlation Analysis of CPET Parameters with Age and BMI

A correlation analysis of all of the measured CPET parameters was performed. We compared the strength of the correlations between the parameters in both the study and control group. We identified a moderate negative correlation of age and peak heart rate in both the study and control groups, with a stronger correlation in the control group. Similarly, we identified moderate strength negative correlations of age with the VO_2_ max, VO_2_/mL/min, VCO_2_ max, MET, P, and P/m. In all cases, the correlations were stronger in the control group in comparison with the study group. Systolic and diastolic blood pressure correlated weakly with BMI in both the study and control group. The resting heart rate correlated weakly with the BMI in the control group. Moderate correlation was identified in the case of BMI and P/m dependence in both groups. The complete results are summarized in Table 4.

### 3.2. Assessment of the Dependence between the Parameter VO_2_ Max mL/kg/min and Post-COVID Disability, or with the Burden of COVID-19

No significant dependence between the parameter VO_2_ max mL/kg/min and post-COVID disability was demonstrated (*p* = 0.412). The Spearman’s correlation analysis did not show a significant relationship between the parameter VO_2_ max mL/kg/min and the severity of COVID-19 (*p* = 0.285). Comparison of the VO_2_ max between individuals with or without pulmonary involvement due to COVID-19 is listed in Table 5, and the correlation analysis is listed in Table 6.

## 4. Discussion

Considering the time since the beginning of the COVID-19 pandemic, there have been very few studies in the literature that have evaluated the cardiorespiratory fitness of people with a past illness and with long COVID syndrome. Ongoing exercise intolerance of unclear cause following COVID-19 infection is well-recognized but poorly understood. The studies differ in terms of the time between the illness and the examination performed as well as the composition of the studies (illnesses with a mild course versus those with a severe course), the variety of consequences after the disease [19], the average age, or the samples were very small [20]. In doing so, cardiorespiratory fitness is an important part of returning to work [21].

From the beginning of the pandemic, the finding of long COVID with cardiological consequences was considered in a high number of patients [22], but gradually, the reported number of complications decreased [23]. This may be because of the less aggressive mutations that are now dominating, or also because of the possible selection bias in earlier studies. In our group, possible cardiac manifestations only occurred in 3% of people. Our group was not statistically significantly different from the control group in the parameter of maximal oxygen consumption (VO_2_ max), which best defines cardiorespiratory fitness. Our findings conflict with those in the study by Raman et al., who found significantly reduced exercise tolerance in post-COVID patients. The study included the evaluation of maximum oxygen consumption and ventilatory efficiency on CPET and six-minute walk distance. However, our study did not involve such a high number of individuals with more pronounced pulmonary involvement. In our study group, persisting significant X-ray changes presented in only 14% of all individuals compared with 60% in the study by Raman [21].

Another study also demonstrated a reduction in the VO_2_ max, but the study was conducted at the time of hospital discharge, so it probably mainly evaluated the condition of the patients [22]. All individuals involved in our study were out-patients, so we could expect a significantly better condition in those cases.

A study by Szekely et al. [24] analyzed the CPET results in a group of 71 patients. They found decreased oxygen consumption measured by VO_2_ max in post-COVID-19 patients compared with the control subjects. The oxygen consumption was also partly decreased due to chronotropic incompetence, as 75% of the post-COVID patients did not reach the expected maximal heart rate. On the other hand, only 8% of the controls had chronotropic incompetence. Moreover, the study found a decreased stroke volume during the exercise in post-COVID individuals [23]. Unfortunately, the study did not provide complete information about the medication of the patients. Similarly, in another study, the post-COVID patients had decreased chronotropic response, represented by decreased maximum heart rate, prolonged heart recovery time, and decreased chronotropic index [25]. In contrast with these results, we only found chronotropic incompetence in one patient. Moreover, the only individual with decreased maximal HR in our study group used beta-blockers, which do not allow us to establish his chronotropic response.

Results of the CPET in post-COVID individuals of our study showed that 60% of them actually had completely normal values and the results were similar to the group of Skørten et al. [26], which also involved patients surviving less severe COVID-19 forms.

In our study group, we did not find a dependence between cardiorespiratory fitness and the severity of lung involvement/course of the disease. However, we admit that the numbers of patients with more severe COVID-19 forms in our study group were very small. There are available studies that have demonstrated the difference between these groups such as the study by Ladlow et al. [27], which compared patients with long COVID with a control group and demonstrated that the patients had persistent functional limitations when compared with active controls, supporting the requirement for ongoing monitoring, rehabilitation, and recovery.

Brown et al. [28] studied exercise tolerance in sixty individuals who were previously admitted to the hospital due to COVID-19 infection. These patients were further divided into those with reduced exercise capacity (i.e., COVIDreduced group) and those with self-reported normal exercise tolerance (i.e., COVIDnormal group). The study also involved the healthy control group. The used method was magnetic resonance-augmented cardiopulmonary exercise testing. The COVIDreduced group had decreased peak oxygen consumption (14.9 mL/min per kg), which was significantly lower compared to the controls (22.3 mL/min per kg of woody-weight; *p* = 0.003) and the COVIDnormal patients (19.1 mL/min per kg; *p* = 0.04). In our study group, the mean VO_2_ max in the post-COVID individuals was 23.3 mL/kg/min, which corresponded to the control group in the study by Brown et al. Interestingly, we found stronger correlations of age with the CPET parameters in the case of the control group compared to the study group. Explanation of this result is possibly due to the increased biological age of post-COVID patients shortly after the infection, as found Mongelli et al. (3%).

We found a statistically significant difference in the power to body-weight ratio (W/kg) between the study and the control group. These findings were similar to those in the study by Rinaldo et al., where COVID-19 survivors showed a mild reduction in their exercise capacity [29]. Study of Jimeno-Almazán et al. [31] found decreased overall cardiopulmonary functions in patients with more severe COVID-19 forms. Individuals with better performance were also significantly less symptomatic. However, it is not clear if this power reduction was actually caused by COVID-19 infection or only by the decondition of the patients. Further studies are needed to confirm or exclude these findings. This is in concordance with the significantly reduced respiratory exchange ratio parameter, which indicates a lower effort in the post-COVID group.

We found significantly lower post-exercise diastolic blood pressure in the post-COVID individuals. This finding can either be a randomly significant result of small group values, or it can be attributed to possible differences between the individuals that do not have a connection with COVID-19.

Few follow-up studies conducted across time points in the first-year post-COVID-19 demonstrated a gradual normalization of VO_2_ max. In a study of 57 COVID-19 survivors aged 56 years on average, 76% of patients demonstrated an impaired VO_2_ max regarding their predicted values at 3 months, and this portion decreased to 60% by 6 months. Notably, at 12 months, 60% of patients still had a decreased VO_2_ max [32]. In contrast, in another follow-up study (*n* = 177, aged 59 years), the VO_2_ max above the predicted threshold was observed in 66% at 3 months compared to 77% of individuals at 12 months [33].

Our study had several limitations. The main limitation that may be considered is the single-center design. However, this may also be an advantage, because all the patients were examined on the same type of machine, which improves the reliability of their interindividual comparison. The second limitation was the relatively low number of involved patients. A possible selection bias may have been present, as individuals with clear pulmonary involvement indicated for specific treatment (mostly corticosteroids) were not included. We only examined individuals with suspicious cardiovascular disease. In addition, we did not perform follow-up CPET in any of the individuals, so we could not establish whether their cardiopulmonary fitness would further improve in time. These patients could provide more decreased CPET values, which may be a possible explanation for some of the discrepancies in our results and the results of the studies above-mentioned. However, it is quite clear that the symptoms of these individuals are related to pulmonary involvement, therefore, we did not perform CPET on them as a very small additional value from such an examination would be expected.

On the other hand, this study is still one of the largest of its type. We can also provide full data for researchers willing to perform meta-analyses.

## 5. Conclusions

Cardiac symptoms and an indication of the CPET occurred in only a small percentage of patients in our study. Cardiorespiratory fitness is one of the most important parameters in the patients’ prognosis. By using CPET, we can quantify the extent of cardiorespiratory system impairment not only in patients with post-COVID syndrome. Differences between post-COVID individuals and the control group might be caused by decondition after the infection, as only their power and RER were significantly lower. Parameters of the respiratory gas exchange were not statistically different between the study and control group. There is a need for further studies that will objectively evaluate the effect of the COVID-19 disease on the patient’s health.

## Figures and Tables

**Table 1:** Basic study group characteristics.

Age	Mean	SD	Median	Minimum	Maximum
All	54.6	14.7	55.0	18	95
Males	56.0	14.5	58.0	18	87
Females	53.4	14.7	53.0	18	95

**Table 2:** Obesity prevalence among groups.

		Controls		Study Group		*p*
		*n*	%	*n*	%	
Obesity prevalence	Males	9	34.6%	11	42.3%	0.569
	Females	18	37.5%	21	43.8%	0.533

**Table 3:** Summary of the comparison of selected parameters between the study and control group.

	Study Group (74)	M (26)	F (48)	Control Group (74)	M (26)	F (48)	Study Group/ Control Group (p)	M (p)	F(p)
Age (mean ± SD)	46.9 ± 10	41 ± 11.5	50.1 ± 7.4	47.2 ± 9.9	41.0 ± 11.6	50.5 ± 6.9	0.801	0.927	0.780
BMI	29.5 ± 7.2	29.5 ± 5.9	29.4 ± 7.8	28.7 ± 5.4	28.9 ± 4.3	28.5 ± 6.0	0.721	0.942	0.720
BMI ≥ 30	32 (43.2%)	11 (42.3%)	21 (43.8%)	27 (36.5%)	9 (34.6%)	18 (37.5%)	0.401	0.569	0.533
BPS rest (mm Hg) (Median, min, max)	130 (100, 180)	135 (115, 155)	128 (100, 180)	135 (100, 170)	140 (120, 160)	133 (100, 170)	0.106	0.167	0.321
BPD rest (mm Hg) (Median, min, max)	80 (60, 110)	85 (70, 110)	80 (60, 105)	85 (50, 110)	90 (70, 100)	80 (50, 110)	0.332	0.763	0.413
HF rest (beat/min) (Median, min, max)	79 (50, 113)	80 (51, 101)	79 (50, 113)	77 (48, 116)	74 (48, 96)	80 (51, 116)	0.721	0.150	0.491
BPS max (mm Hg) (Median, min, max)	180 (145, 245)	190 (145, 230)	173 (145, 245)	185 (140, 265)	193 (160, 265)	180 (140, 230)	0.202	0.118	0.446
BPD max (mm Hg) (Median, min, max)	80 (50, 125)	80 (50, 110)	80 (50, 125)	90 (20, 120)	90 (60, 120)	90 (20, 120)	**0.003**	**0.049**	**0.023**
HF max (beat/min) (Median, min, max)	161 (90, 194)	170 (132, 194)	159 (90, 186)	161 (113, 202)	170 (113, 202)	160 (120, 191)	0.558	0.749	0.605
VO_2_ max (mL) (Median, min, max)	1987 (998, 4390)	2870 (1905, 4390)	1708 (998, 2592)	2124 (1119, 5791)	3108 (1608, 5791)	1842 (1119, 3123)	0.272	0.288	0.142
VO_2_ max mL/kg/min (Median, min, max)	23.3 (12.9, 65.5)	28.6 (15.2, 65.5)	22.0 (12.9, 39.1)	26.2 (14.8, 57.9)	32.5 (16.3, 57.9)	23.6 (14.8, 48.8)	0.082	0.570	0.070
VO_2_ max mL/kg/min in population norm	45 (60.8%)	15 (57.7%)	30 (62.5%)	49 (66.2%)	19 (73.1%)	30 (62.5%)	0.495	0.244	1.000
RER (Median, min. max)	1.16 (1.05, 1.4)	1.18 (1.05. 1.32)	1.16 (1.05, 1.40)	1.2 (1.05, 1.69)	1.2 (1.05, 1.69)	1.22 (1.09, 1.63)	**0.0003**	0.077	**0.001**
VE/VCO_2_ slope (Median, min, max)	32.0 (20.4, 46.0)	30.3 (22.8, 44.4)	33.0 (20.4, 46.0)	30.7 (19.2, 66.8)	28.8 (19.4, 66.8)	32.4 (19.2, 52.5)	0.168	0.415	0.260
MET (Median, min, max)	7.2 (3.6, 13.8)	8.4 (5.1, 13.8)	6.5 (3.6, 12.2)	7.8 (4.1, 20.0)	9.3 (4.7, 20.0)	7.3 (4.1, 13.4)	0.096	0.301	0.098
P (W) (Median, min, max)	165 (85, 342)	255 (180, 342)	143 (85, 231)	190 (100, 411)	296 (170, 411)	155 (100, 246)	**0.048**	0.057	**0.008**
P/m (W/kg) (Median, min, max)	2.1 (0.85, 4.0)	2.68 (1.6, 4.0)	1.9 (0.85, 3.25)	2.22 (1.2, 4.5)	3.09 (1.7, 4.5)	2.01 (1.2, 4.2)	**0.039**	0.237	**0.048**

Abbreviations: M = males, F = females, BMI = body mass index, BPS = beats per second, HF = heart frequency, BPS = blood pressure systolic, BPD = blood pressure diastolic, VO_2_ max = maximal oxygen consumption per minute and kilogram of body weight, RER = respiratory exchange ratio, m = weight, VE = maximal minute ventilation, VCO_2_ = carbon dioxide excretion, MET = metabolic equivalent of task, P = power, W = watts, W/kg. Bold *p*-values = statistically significant.

**Table 4:** Correlations of the CPET parameters with age and BMI in the control and study group.

Age Correlations (Controls)	BPS Rest	BPD Rest	HR Rest	HR Max	BPS Max	BPD Max	VO_2_ Max	VO_2_ Max mL/kg/min	VCO_2_ Max	RER	VE/ VCO_2_	MET	*p*	*p*/m
Correlation Coefficient	0.011	0.090	0.084	**−0.669**	0.034	0.114	**−0.607**	**−0.519**	**−0.658**	−0.174	0.116	**−0.495**	**−0.634**	**−0.521**
*p*	0.924	0.444	0.475	**<0.0001**	0.771	0.334	**<0.0001**	**<0.0001**	**<0.0001**	0.138	0.341	**<0.0001**	**<0.0001**	**<0.0001**
Age correlations(study group)														
Correlation Coefficient	0.040	−0.151	−0.032	**−0.535**	−0.042	0.039	**−0.492**	**−0.346**	**−0.499**	−0.146	0.032	**−0.260**	**−0.486**	**−0.339**
*p*	0.733	0.199	0.787	**<0.0001**	0.724	0.741	**<0.0001**	**0.003**	**<0.0001**	0.215	0.791	**0.025**	**<0.0001**	**0.003**
BMI correlations(controls)														
Correlation Coefficient	**0.431**	**0.370**	**0.298**	0.010	**0.384**	**0.275**	0.149	**−0.435**	0.157	−0.154	−0.027	**−0.319**	0.076	**−0.491**
*p*	**0.0001**	**0.001**	**0.010**	0.931	**0.001**	**0.018**	0.204	**0.0001**	0.182	0.191	0.821	**0.006**	0.519	**<0.0001**
BMI correlations(study group)														
Correlation Coefficient	**0.398**	**0.323**	0.078	−0.096	0.216	**0.405**	0.146	**−0.433**	0.077	**−0.354**	−0.065	**−0.450**	0.080	**−0.507**
*p*	**0.0004**	**0.005**	0.511	0.417	0.065	**0.0003**	0.214	**0.0001**	0.533	**0.002**	0.588	**0.0001**	0.498	**<0.0001**

Bold *p*-values = statistically significant.

**Table 5:** Comparison of oxygen consumption in individuals with or without pulmonary involvement due to COVID-19.

		Pulmonary Post-COVID Involvement		*p*
		No	Yes	
VO_2_ max mL/kg/min	Median	24.4	22.4	0.412
	Minimum	12.9	12.9	
	Maximum	46.9	65.5	

**Table 6:** Correlation of the CPET parameters with COVID-19 severity.

COVID-19 Severity vs.	BPS Rest	BPD Rest	HR Rest	HR Max	BPS Max	BPD Max	VO_2_ Max	VO_2_ Max mL/kg/min	VCO_2_ Max	RER	VE/VCO_2_	MET	*p*	*p*/m
Correlation Coefficient	0.115	0.001	−0.189	−0.302	0.137	0.074	0.096	0.218	0.161	0.382	−0.233	0.210	0.083	0.121
*p*	0.577	0.996	0.356	0.133	0.503	0.718	0.640	0.285	0.453	0.054	0.262	0.303	0.693	0.563

## Data Availability

The data presented in this study are available on request from the corresponding author. The data are not publicly available due to privacy of the patients.
